# Prenatal exposure to tetrachloroethylene-contaminated drinking water and the risk of congenital anomalies: a retrospective cohort study

**DOI:** 10.1186/1476-069X-8-44

**Published:** 2009-09-24

**Authors:** Ann Aschengrau, Janice M Weinberg, Patricia A Janulewicz, Lisa G Gallagher, Michael R Winter, Veronica M Vieira, Thomas F Webster, David M Ozonoff

**Affiliations:** 1 Department of Epidemiology, Boston University School of Public Health, Talbot 3E, 715 Albany Street, Boston, MA 02118, USA; 2 Department of Biostatistics, Boston University School of Public Health, Crosstown, 715 Albany Street, Boston, MA 02118, USA; 3 Department of Environmental Health, Boston University School of Public Health, Talbot 4W, 715 Albany Street, Boston, MA 02118, USA; 4 Data Coordinating Center, Boston University School of Public Health, Crosstown, 715 Albany Street, Boston MA 02118, USA

## Abstract

**Background:**

Prior animal and human studies of prenatal exposure to solvents including tetrachloroethylene (PCE) have shown increases in the risk of certain congenital anomalies among exposed offspring.

**Objectives:**

This retrospective cohort study examined whether PCE contamination of public drinking water supplies in Massachusetts influenced the occurrence of congenital anomalies among children whose mothers were exposed around the time of conception.

**Methods:**

The study included 1,658 children whose mothers were exposed to PCE-contaminated drinking water and a comparable group of 2,999 children of unexposed mothers. Mothers completed a self-administered questionnaire to gather information on all of their prior births, including the presence of anomalies, residential histories and confounding variables. PCE exposure was estimated using EPANET water distribution system modeling software that incorporated a fate and transport model.

**Results:**

Children whose mothers had high exposure levels around the time of conception had an increased risk of congenital anomalies. The adjusted odds ratio of all anomalies combined among children with prenatal exposure in the uppermost quartile was 1.5 (95% CI: 0.9, 2.5). No meaningful increases in the risk were seen for lower exposure levels. Increases were also observed in the risk of neural tube defects (OR: 3.5, 95% CI: 0.8, 14.0) and oral clefts (OR 3.2, 95% CI: 0.7, 15.0) among offspring with any prenatal exposure.

**Conclusion:**

The results of this study suggest that the risk of certain congenital anomalies is increased among the offspring of women who were exposed to PCE-contaminated drinking water around the time of conception. Because these results are limited by the small number of children with congenital anomalies that were based on maternal reports, a follow-up investigation should be conducted with a larger number of affected children who are identified by independent records.

## Background

In 1980 New England government officials discovered that PCE (perchloroethylene, tetrachloroethylene) was leaching into public drinking water supplies from the inner vinyl lining (VL) of asbestos cement (AC) water distribution pipes. The vinyl liner had been introduced in the late 1960s to solve taste and odor problems in some sections of the distribution system. The liner had been painted onto the inner surface of the pipe in a slurry of vinyl toluene resin (Piccotex,™ Johns-Manville Corporation, Denver, CO) and PCE. After a 48 hour drying period, the pipes were shipped to the towns for installation [1]. Because PCE is a volatile solvent, the manufacturer assumed that most would evaporate by the time the pipes were installed. However, more than a decade elapsed before officials discovered that high levels of PCE remained in the liner and were slowly discharging into the public drinking water supplies.

**Figure 1:**
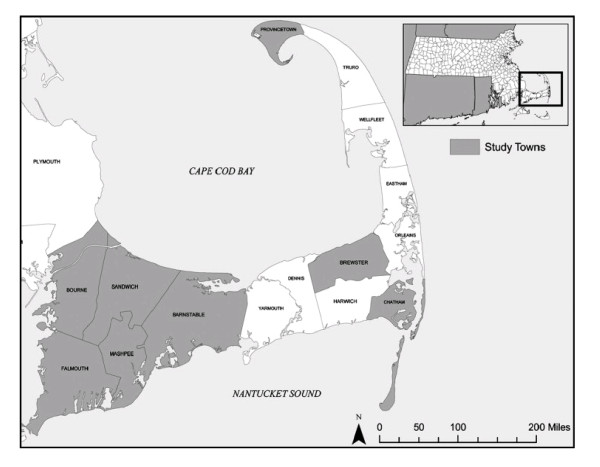
**Geographic location of Cape Cod study area**. Cape Cod is located in Massachusetts in the northeastern area of the United States. Study towns are highlighted.

An investigation revealed that approximately 660 miles of VL/AC pipes were installed in Massachusetts [2]. A sizeable portion had been installed in the Cape Cod region (Figure 1). Because the lined pipes had been used to replace existing pipes and to extend the water system, the pattern of contamination was quite irregular. Adjacent streets and even adjacent houses had different supply pipes, leading to a "vast natural experiment" reminiscent of John Snow's cholera investigation in 1854 London [3].

PCE levels in residential areas in the Cape Cod town of Falmouth ranged from undetectable to 80 μg/L in water pipes along main streets with high water flow and from 1,600 to 7,750 μg/L in water pipes along dead end streets with low water flow [1]. Currently, U.S. E.P.A. drinking water regulations set PCE's maximum contaminant level at 5 μg/L [4]. The main exposure routes for PCE-contaminated drinking water are ingestion, as well as inhalation, and dermal exposure during showering and bathing.

During this time period, concentrations of other measured drinking water contaminants were low [5]. Because it was too costly to replace the VL/AC pipes, officials instituted a flushing and bleeding program in the most problematic areas to reduce levels below 40 μg/L, the suggested action guide in 1980 [1].

While health concerns regarding PCE have been based mainly on its carcinogenicity [6], animal experiments also suggest an adverse effect of prenatal exposure to PCE, the closely related solvent trichloroethylene (TCE), and their metabolite trichloroacetic acid (TCA) on the risk of congenital anomalies. In particular, an increased prevalence of several malformations, including cardiovascular, musculoskeletal, central nervous system and ocular anomalies, have been observed among chicks and rats over a wide range of prenatal exposure levels [e.g., [7,8]].

Several epidemiological studies have also found associations between prenatal exposure to organic solvents and the risk of congenital anomalies. A meta-analysis of studies on maternal occupational exposure to solvents found a statistically significant 60% increased risk of major malformations. While studies among female dry cleaners exposed to PCE have found no increased risk of congenital anomalies, small sample sizes limited their statistical power. Studies of women exposed to contaminated drinking water have reported increases in the risk of cardiac and central nervous system anomalies, as well as oral clefts [e.g., [9,10]].

We undertook a population-based retrospective cohort study to examine the influence of maternal exposure to PCE contaminated drinking water on a variety of pregnancy and developmental outcomes, including low birth weight, prematurity and learning disabilities [11,12]. The current report focuses on the risk of congenital anomalies, using the reproductive histories reported by mothers in the study.

## Methods

### Selection of Study Population

Women were eligible for the study if they gave birth to a child (termed "index child") during 1969-1983 while they were living in a Cape Cod town with some VL/AC water distribution pipes. Eight towns met this condition, including Barnstable, Bourne, Falmouth, Mashpee, Sandwich, Brewster, Chatham, and Provincetown, Massachusetts (Figure 1). Considerable population exposure took place in these towns during this 15-year time period. The extent of VL/AC pipes in these towns ranged from one mile in Mashpee to 50 miles each in Falmouth and Sandwich [13]. Eligible mothers were identified by manually reviewing over 13,000 Massachusetts birth certificates and cross-matching the maternal address on the certificate against address and water distribution data collected from local water companies which included the location, installation year, and diameter of all VL/AC water pipes in the Cape Cod area.

Two groups of women were selected: (1) mothers who were exposed to PCE-contaminated drinking water when the "index" child was born, and (2) mothers who were unexposed when the "index" child was born. Mothers were initially designated as "exposed" because the birth residence was either directly adjoining a VL/AC pipe or because the birth residence was directly adjoining a pipe connected to a VL/AC pipe and the only possible water flow to their residence was through the VL/AC pipe. This initial designation was based on a visual inspection of maps depicting the pipe distribution network in the immediate vicinity of the maternal address at the time of the birth. Children received initial exposure designations by a member of our research team who was familiar with the water distribution systems on Cape Cod. The initial "exposed" group included 1,492 mothers who gave birth to 1,862 singletons and 24 sets of twins.

A comparison group of mothers initially designated as "unexposed" was randomly selected from the remaining resident women who gave birth during this period. "Unexposed" mothers were frequency matched to "exposed" mothers on the month and year of birth of their index child. The initial "unexposed" group included 1,704 mothers who gave birth to 1,853 singletons and 37 sets of twins or triplets. The initial exposure status of each birth was considered tentative until survey data on private well use were available and more extensive exposure assessments, as described below, were completed.

Birth certificates were reviewed to obtain information on family members including the full names of the index child and parents; the child's dates of birth; the parents' ages and educational levels; the date of the mother's last menstrual period; the adequacy of prenatal care; and the index birth child's birth weight and gestational age.

The study was approved by the Institutional Review Boards of the Massachusetts Department of Public Health and Boston University Medical Center, and by the 24A/B/11B Review Committee at the Massachusetts Department of Public Health.

### Follow-Up and Enrollment of Study Subjects

Follow-up and enrollment of mothers occurred during 2002-2003. Women were traced to obtain their current addresses and telephone numbers. Tracing resources included Massachusetts Resident's Lists; death, marriage, divorce, credit bureau and alumni records; telephone books, directory assistance, and the Internet White Pages. Letters were sent to all traced mothers describing the general purpose of the study and requesting that they complete a self-administered questionnaire. Two follow-up letters were sent to non-respondents, and women who did not respond to these letters were phoned. As described in Table 1, 8.4% of the selected population could not be located, 18.2% were located but never responded to any of our contact attempts, and 8.9% refused to participate. Another 0.5% of women were considered ineligible because the birth certificate address was determined to be a temporary residence or because they were deceased. These percentages were similar for both "exposed" and "unexposed" women.

**Table 1:** Selection and Enrollment According to Women's PCE Exposure Status, Cape Cod, Massachusetts

	Initial Exposure Status*		
	**Number Exposed**	**Number Unexposed**	**Total Number**

Selected	1,492	1,704	3,196

Excluded during Enrollment			

Never located	132	136	268

No response	245	336	581

Ineligible or Deceased	7	8	15

Refusal	149	137	286

Returned Questionnaire	959	1,087	2,046

% of selected	64.3%	63.8%	64.0%

% of located	70.5%	69.3%	69.9%

*The exposure status was based on the initial visual assessment. See text for details.

We conducted analyses comparing birth certificate data on birth weight, gestational duration, and demographic characteristics among children of participants and non-participants. The mean gestational duration among children of non-participants was similar to that of participants (40.1 vs. 40.2 weeks for non-participants vs. participants, respectively); however, the mean birth weight among children of non-participants was about 100 grams lighter (3,381 vs. 3,483 grams for non-participants vs. participants, respectively). The race and birth year of children of non-participants (96.2% white, 54.7% born during 1979-1983) were similar to that of the participants (96.2% white, 56.0% born during 1979-1983); however, non-participating mothers were younger (mean age 26.0 vs. 27.5 years), less educated (11.3% did not graduate from high school vs. 3.6%) and had more prior births (51.1% had three or more prior births vs. 24.3%). These differences were present for both exposed and unexposed non-participants. For example, 11.2% vs. 11.4% of exposed and unexposed non-participants did not graduate high school. (The exposure status of non-participants was based on the initial visual designation.)

Self-administered questionnaires were sent to all successfully traced mothers to gather information on their entire reproductive history, including the presence of congenital anomalies among all index and non-index births. In addition, the questionnaires gathered information on maternal demographic characteristics including race and educational level; data on smoking, alcohol intake, caffeine consumption, weight gain and obstetrical complications during each pregnancy; medical conditions such as diabetes and hypertension; occupational exposure to solvents; use of solvent-based spot removers, professional and self-service dry cleaning. In addition, information was collected on the family's residences from 1969 though 1990; and the proximity of any residences to dry cleaning establishments. The residential history included the calendar years of residence, the exact street address, and nearest cross-street for all Cape Cod residences. While we attempted to collect information on the mother's water consumption and bathing habits at these residences, this information could not be recalled by a sizable portion of the mothers.

Following receipt of a completed questionnaire, we requested permission to review the prenatal and delivery records of the last-born "index" child from each participating mother. About 250 mothers agreed to release these records and the records for 60 mothers were obtained. The remainder could not be located by the delivery hospital or obstetrician; all of these records were 20-30 years old. The reproductive histories and related information in these medical records were compared to that reported by mothers in the self-administered questionnaires. We also compared reproductive history data reported in the questionnaires with that on the birth certificates. The latter analyses were conducted among all births occurring in Massachusetts (n = 2,490), which was 53% of reported births. Variables examined for concordance between the latter two sources included birth weight and gestational duration, and smoking and alcohol use during pregnancy. We were unable to validate maternal reports of congenital anomalies against pediatric records because these records could not be obtained. Available delivery records and birth certificates were considered a poor source of information on congenital anomalies.

### Congenital Anomaly Review and Coding

All maternal reports of congenital anomalies were reviewed by two individuals (AA and PAJ) with knowledge of teratology and in consultation with a pediatrician. We considered for inclusion in our analysis only diagnoses currently regarded congenital malformations by the Metropolitan Atlanta Congenital Defects Program (MACDP) [14]. We coded the reported anomalies according to these guidelines without knowledge of the exposure status of the child. Anomalies were categorized into major and minor malformations and organ system groups; and, if the numbers were sufficient, into specific diagnostic categories. Anomaly groups included central nervous, cardiovascular, respiratory, gastrointestinal, genitourinary tract, and musculoskeletal systems; eye; ear, face and neck; chromosomal; and other and unspecified anomalies. Children with neural tube defects, oral clefts and hypospadias were also examined separately.

### Geocoding of Residential Addresses

All residential addresses on Cape Cod reported on the questionnaires were geocoded to a latitude and longitude using ArcGIS 8.1. Whenever possible, we assigned each address to a parcel of land. Addresses that could not be geocoded initially were researched using county deeds, assessors' maps, town voter registration lists, and internet resources. Addresses that could not be geocoded to a particular parcel using information from the questionnaire and the ancillary sources were geocoded to the closest parcel address by street number. If a street number was unavailable, the address was geocoded to the middle of the street, if the street was less than a mile long, or to the intersection of the address with the cross-street given in the survey, if the street was a mile or longer. Approximately 97% of reported addresses were successfully geocoded. The remainder could not be geocoded because of insufficient information. All geocoding was conducted without knowledge of the exposure or outcome status.

### PCE Exposure Assessment

We assigned an initial exposure designation to each index child after visually inspecting maps of the pipe distribution network in the immediate vicinity of the mother's address when the index child was born. To determine the final exposure designation for all index and non-index births, we used a leaching and transport model to estimate the mass of PCE that was delivered to each residence during the relevant exposure period. The model, developed by Webler and Brown for our previous epidemiological studies [15,16], estimates the amount of PCE entering the drinking water using the initial amount of PCE in the pipe liner, the pipe's age, and the leaching rate of PCE from the liner into the water. The pipe's initial stock of PCE is based on the size of the pipe (i.e., diameter, length) and information from the pipe manufacturer on the application of the liner. The leaching rate of PCE, which declines with time, was determined from experiments conducted by Demond [1]. The rate calculation assumes that there is a finite amount of PCE in the liner, a uniform distribution of the liner along the pipe length, and a uniform starting amount of PCE in the liner.

The algorithm also requires an estimate of water flow, which is a function of the distribution pipe configuration (geometry) and number of water users. The flow rate calculation is used to determine the residence time of water in each pipe segment and to determine the direction of PCE-contaminated water flow through the system of connected pipe segments. In our early studies, we simplified the effect of pipe configuration on flow by considering combinations of generic cases such as dead-ends, circles, circles with taps and in-line pipes [16]. For the current study, we incorporated the Webler and Brown algorithm into EPANET water distribution system modeling software in order to improve our estimate of water flow and direction. The EPANET software, developed by the U.S. Environmental Protection Agency has been applied in several other epidemiological investigations [17-21]. The software simulates the instantaneous flow of water throughout a town's entire public water distribution system.

Using GIS maps of subject residences and a town's distribution system, we created a schematic depicting the water source locations; pipe characteristics indicating length, diameter and composition; and nodes, the points along the pipe where water consumption occurs. Information on the locations, installation dates and diameters of all VL/AC pipes in the public water supply were obtained from maps and other documents provided by fifteen local water departments and the Massachusetts Department of Environmental Protection (DEP). The available information reflected the water system conditions around 1980, and so we chose this year as representative of the water flow during the entire study period.

Using the schematic, we assigned each residence to the closest node on the distribution system. We assumed that all users on the network drew the same amount of water during a woman's residence because the study area was mainly comprised of residences. Typical values for other parameters were assumed in the absence of historical data and when they had low level of variability. For example, seasonal variation in water temperature was not taken into account, and node elevation is dependent on topography which is essentially constant in the Cape Cod region. We also assumed that water sources did not change over the study period. The distribution systems that were in place in the 1960s and early 1970s remained generally unchanged until population growth required some systems to expand and add sources in the 1980s.

The EPANET software incorporated these data to simulate the instantaneous flow of water through the thousands of pipe segments in each town's network and to estimate the mass of PCE in grams delivered to each node and all women's residences associated with the node. We were able to calculate only annual PCE exposures because we knew only the move-in and pipe installation years. We estimated the average monthly PCE exposure during the prenatal period of each birth by dividing the annual mass of PCE that entered an exposed residence during the year of the last menstrual period (LMP) by twelve. The first trimester was completed during the same year as the LMP for 85% of study pregnancies, and the Pearson correlation coefficient between annual PCE exposure levels during the LMP and first trimester years was 0.96 (p < 0.0001). The LMP year was estimated from questionnaire or birth certificate data (if questionnaire data were missing) on gestational duration and birth date. The LMP could not be estimated for 226 births that were excluded from the analysis.

We estimated PCE exposure levels only for children whose mothers had complete geocoded residential histories. A total of 534 children had mothers with inadequate residential histories and so were excluded from the analysis. All children whose mothers reported using a private well for their drinking water supply throughout their entire residence at a Cape Cod address or who lived at an address in a Cape Cod town without any VL/AC pipes were assumed to have no PCE exposure. This was considered a reasonable assumption because available records from this geographic area and time period indicated little or no PCE contamination of these water sources.

### Statistical Analysis

A total of 4,657 children, including both index and non-index children, were included in the final analysis. Births occurring after 1990 (n = 108), that were missing prenatal information (n = 226), from multiple pregnancies (n = 129), and among mothers who were exposed to known teratogen(s) during pregnancy (n = 58), smoked marijuana on a weekly or daily basis (n = 33), or drank seven or more alcoholic drinks per week during pregnancy (n = 89) were excluded.

The analysis compared the occurrence of congenital anomalies among children with and without prenatal exposure. We examined all congenital anomalies combined, all major congenital anomalies combined, organ system groups, and specific diagnostic categories. Children with more than one anomaly diagnosis could contribute to more than one organ system or diagnostic group. The odds ratio was used to estimate the strength of the association between PCE exposure and the occurrence of an anomaly. Odds ratios were calculated only if there were at least three exposed cases. Ninety-five percent confidence intervals were used to assess the precision of the odds ratios.

We used a locally weighted regression smoother (LOESS) to examine the shape of the relationship between exposure and outcome under study [22]. These analyses did not identify any natural cut points, and so we arbitrarily divided the exposure measure into quartiles. In addition, we dichotomized the average monthly prenatal exposure at the level corresponding to an average drinking water concentration of 40 ug/L, the suggested action guide when the pollution was discovered in 1980.

Generalized estimating equation (GEE) analyses were conducted to account for non-independent outcomes arising from several children born to the same woman [23,24]. Eighty-eight percent of the mothers had two or more children during the study period. The logit link was used while assuming equal correlation between birth outcomes from the same mother. Unadjusted GEE analyses were attempted if there were at least three exposed cases; in some instances, the small number of subjects prevented the model from converging.

Adjusted GEE analyses were also conducted to control for confounding variables. Covariates considered for these analyses were known risk factors for congenital anomalies or non-drinking water sources of solvent exposure. These variables included calendar year of birth; gender of the child; maternal race, age, and educational level; paternal age and occupation; gravidity, number of prior live births, prior child with a congenital anomaly (before the exposure or a comparable index year for the unexposed), and number of prior pregnancy losses; maternal alcoholic beverage consumption and cigarette smoking during the first trimester; marijuana and multivitamin use during pregnancy; maternal history of diabetes, hypertension, pre-eclampsia, DES exposure, prenatal infection; maternal history of occupational exposure to solvents, lead, and anesthetic gases, solvent-based spot removers and dry cleaning; and prenatal residence in Falmouth, the only Cape Cod town with a chlorinated surface water supply.

When all anomalies were considered as a single group, each of these variables was entered into the GEE model one at a time and the crude and adjusted GEE results were compared. Because none of these variables resulted in meaningful changes in the crude results, multivariate GEE odds ratios were adjusted simultaneously only for maternal and paternal age. We also attempted to control for one confounder at a time in subgroups with at least 20 affected children; these included cardiac, gastrointestinal, genitourinary and musculoskeletal defects. In some instances, the small number of subjects prevented the model from converging. However, when the model did converge, the results (given below) indicated that there was little or no confounding.

Lastly, to determine if recall bias was present, we conducted analyses comparing the mothers' self-assessed exposures with the independent EPANET assessment.

## Results

Following the EPANET exposure assessment, there were 61 children with congenital anomalies among 1,658 children with some prenatal PCE exposure and 95 children with congenital anomalies among 2,999 children with no prenatal PCE exposure. The corresponding prevalence proportions per 1,000 births were 3.7 and 3.2, respectively. Many characteristics of the exposed and unexposed groups were similar (Table 2). For example, mothers in both groups were predominantly white, and comparable proportions had medical conditions, prenatal multivitamin use, and exposure to non-drinking water sources of solvents. However, there were also many differences between the groups. Due to the timing of the PCE contamination, exposed mothers were more likely to give birth in later calendar years. Exposed mothers and fathers were older than unexposed parents, and exposed mothers were less likely to smoke cigarettes during the first trimester. Exposed children with anomalies were also more likely to be male and have mothers with high educational levels and prior pregnancy losses, and alcoholic beverage consumption during the first trimester.

**Table 2:** Distribution of Selected Characteristics of Parents and Children by Prenatal PCE Exposure and Outcome Status

**Characteristic**^+^	Exposed With Anomalies		Exposed Without Anomalies		Unexposed With Anomalies		Unexposed Without Anomalies	
	n	%	n	%	n	%	n	%
Year of birth								

Before 1968	0	0.0	0	0.0	12	12.6	313	10.8

1968-1974	10	16.4	239	15.0	28	29.5	770	26.5

1975-1980	27	44.3	684	42.8	29	30.5	1014	34.9

After 1980	24	39.3	674	42.2	26	27.4	807	27.8

Gender of infant								

Male	36	59.0	791	49.7	51	53.7	1454	50.8

Female	25	41.0	801	50.3	44	46.3	1407	49.2

Maternal age (n, mean (sd))	61	28.4 (5.2)	1588	27.6 (4.6)	95	25.1 (4.6)	2886	26.0 (4.9)

Paternal age (n, mean (sd))	61	31.9 (6.6)	1583	30.8 (5.9)	94	27.3 (6.2)	2849	28.9 (5.8)

% White Race	60	98.4	1522	95.5	91	95.8	2774	95.9

Maternal educational level								

High school graduate or less	7	11.5	331	20.8	22	23.2	667	23.0

Some college	16	26.2	561	35.2	35	36.8	996	34.4

Four year college grad or higher	38	62.3	701	44.0	38	40.0	1234	42.6

Paternal occupation								

White collar	37	61.7	809	51.6	47	51.1	1315	45.9

Blue collar	15	25.0	522	33.3	26	28.3	988	34.4

Other	8	13.3	234	15.0	19	20.7	565	19.7

Gravidity								

1	17	27.9	388	24.3	36	37.9	1073	36.9

2	17	27.9	561	35.1	31	32.6	846	29.1

3+	27	44.3	648	40.6	28	29.5	985	33.9

Prior live births								

0	22	36.1	491	30.8	43	45.3	1240	42.7

1	24	39.3	615	38.5	31	32.6	934	32.2

2+	15	24.6	490	30.7	21	22.1	729	25.1

History of prior pregnancy loss	20	32.8	273	17.1	13	13.7	436	15.0

Maternal cigarette smoking during first trimester								

11+ cigarettes a day	7	11.5	220	13.8	16	17.0	502	17.5

10 cigarettes or fewer a day	7	11.5	168	10.6	9	9.6	356	12.4

None	47	77.0	1202	75.6	69	73.4	2018	70.2

Maternal alcohol consumption during first trimester^&^								

1+ drinks a week	13	21.3	175	11.0	8	8.5	337	11.7

1-3 drinks a month	20	32.8	389	24.5	20	21.3	714	24.9

None	28	45.9	1022	64.4	66	70.2	1820	63.4

Maternal multivitamin use during pregnancy	59	96.7	1492	94.4	88	94.6	2653	93.1

Maternal occupational exposure to solvents before or during pregnancy	8	13.3	190	12.1	7	7.6	296	10.3

Maternal residential proximity to dry cleaning establishment	0	0.0	7	0.7	0	0.0	8	0.3

Maternal use of solvent-based spot removers: occasional or frequent use	16	27.1	342	21.8	30	31.9	614	21.7

Maternal use of professional dry cleaning: use > = once per month	17	27.9	468	29.7	31	33.7	843	30.4

Maternal use of self-service dry cleaning: ever use	12	19.7	209	13.4	19	20.4	460	16.3

*Prenatal exposure based on final exposure designation.

^+^Individuals with missing data are excluded from the percentages.

^&^Heavy users of alcohol were excluded from the analysis

Nearly 39% of mothers could not recall their water consumption or bathing patterns during the study pregnancy (data not shown). However, among women who could recall this information, the proportions who drank bottled water (about 22%), consumed more than four glasses of tap water per day (about 51%), and took long showers (about 23%) were similar across the exposed and unexposed groups.

There was a wide distribution of PCE exposure levels encompassing several orders of magnitude in the exposed group. Average monthly PCE exposure levels during the LMP year ranged from 9.6E-05 to 131.8 grams. The 25^th^, 50^th^, 75^th ^and 90^th ^percentiles were 0.1, 0.6, 2.3, and 6.4 grams, respectively. As previously described, the exposure measures were based on the mass of PCE delivered to a home in each calendar year. The annual mass of PCE entering a home was diluted in an estimated 90,000 gallons of water, the annual usage of average households in Massachusetts [25], and only a small portion of this water was directly consumed by the subjects. Using this annual estimate of household water use, we converted the PCE mass delivered to a home during pregnancy to average annual point concentrations and estimated that the PCE concentrations in the water entering the homes ranged from less than 1 ug/L to 5,197 ug/L. These concentrations are consistent with actual water sampling data from the time period [1].

The crude and unadjusted GEE odds ratios for all congenital anomalies combined were 1.2 (95% CI: 0.8, 1.6) and 1.1 (95% CI: 0.8, 1.6), respectively, among children with any prenatal PCE exposure (Table 3). These odds ratios were virtually unchanged when maternal and paternal age were controlled simultaneously (multivariate GEE OR: 1.2, 95% CI: 0.8, 1.7, Table 3) and when other confounders, including calendar year of birth; mother's educational level, cigarette smoking, alcoholic beverage consumption, and prior pregnancy losses; and child's gender were controlled one at a time (GEE ORs: 1.1-1.2, data not presented in Table 3). These results were unchanged when only major malformations were examined.

**Table 3:** Frequencies, Crude, Unadjusted and Adjusted GEE Odds Ratios (OR) and 95% Confidence Intervals (CI) for All Congenital Anomalies Combined by Prenatal PCE Exposure

	Number with Anomalies	Total Number	Crude OR (95% CI)	Unadjusted GEE OR (95% CI)	Multivariate* GEE OR (95% CI)
Any Exposure					

>0	61	1658	1.2 (0.8,1.6)	1.1 (0.8,1.6)	1.2 (0.8,1.7)

0 (referent)	95	2999	1.0 (--------)	1.0 (--------)	1.0 (--------)

Exposure Categorized by 1980 Action Level of 40 ug/L					

>1.136 g (40 ug/L)	26	605	1.4 (0.9,2.1)	1.4 (0.9,2.2)	1.4 (0.9,2.2)

>0 - <= 1.136 g	35	1053	1.1 (0.7,1.6)	1.0 (0.6,1.5)	1.0 (0.7,1.6)

0 (Referent)	95	2999	1.0 (--------)	1.0 (--------)	1.0 (---------)

Exposure Categorized in Quartiles					

>75^th ^p'tile	19	415	1.5 (0.9, 2.4)	1.5 (0.9, 2.4)	1.5 (0.9, 2.5)

50th - <75^th ^p'ctile	14	414	1.1 (0.6, 1.9)	1.0 (0.6, 1.9)	1.1 (0.6, 2.0)

25th - <50^th ^p'ctile	15	415	1.1 (0.7, 2.0)	1.1 (0.6, 2.0)	1.1 (0.6, 2.0)

>0 - <25th p'ctile	13	414	1.0 (0.5, 1.8)	0.9 (0.5, 1.8)	1.0 (0.5, 1.9)

0 (referent)	95	2999	1.0 (---------)	1.0 (---------)	1.0 (---------)

*Adjusted for maternal and paternal age

The parental-age adjusted GEE odds ratio for all anomalies was elevated by 40% (95% CI: 0.9-2.2) among children whose average monthly prenatal exposure was greater than 1.136 grams, the cut point corresponding to an average drinking water concentration of 40 ug/L, and elevated by 50% among children whose average monthly prenatal exposure was > = 75^th ^percentile (95% CI: 0.9-2.5) (Table 3). The 75^th ^percentile corresponded to an average monthly prenatal exposure of 2.3 grams. No meaningful increases in risk were seen for lower exposure levels. Again, these results were unchanged when only major malformations were included.

When organ system and diagnostic groups were examined (Table 4), we found large increases in the odds ratios for neural tube defects (GEE OR 3.5, 95% CI: 0.8-14.0) and oral clefts (GEE OR 3.2, 95% CI 0.7-15.0); and modest increases in the odds ratios for gastrointestinal (GEE OR 1.8, 95% CI: 0.7-4.4) and genitourinary malformations (GEE OR 1.6, 95% CI: 0.6-3.8), including hypospadias (GEE OR: 1.4, 95% CI: 0.4-5.4); and chromosomal malformations (GEE OR: 1.4, 95% CI: 0.3-6.1) among children with any prenatal PCE exposure (Table 4). No meaningful increases in odds ratios were seen for cardiac and musculoskeletal malformations, and there were too few exposed cases to estimate odds ratios for eye, ear, respiratory, and other malformations (Table 4).

**Table 4:** Frequencies, Odds Ratios (ORs), and 95% Confidence Intervals (CI) for Congenital Anomaly Categories by Prenatal PCE Exposure

Anomaly Category	Exposure Category	Number with Anomaly	Total Number	Crude OR (95% CI)	Unadjusted GEE OR* (95% CI)
					

Central Nervous System	Any	7	1604	3.2 (0.9,11)	3.1 (0.9,11)

	>1.136 g (40 ug/L)	1	580	--------	--------

	>0 - <= 1.136 g	6	1024	4.3 (1.2,15)	4.1 (1.1,16)

	0 (Referent)	4	2908	1.0 (--------)	1.0 (--------)

					

Neural Tube Defects+	Any	6	1603	3.6 (0.9, 15)	3.5 (0.8, 14)

	>1.136 g (40 ug/L)	0	579	---------	---------

	>0 - <= 1.136 g	6	1024	5.7 (1.4, 23)	---------

	0 (Referent)	3	2904	1.0 (-------)	1.0 (-------)

					

Cardiac	Any	9	1606	0.9 (0.4,2.0)	0.9 (0.4,2.0)

	>1.136 g (40 ug/L)	4	583	1.1 (0.4,3.3)	1.1 (0.4,3.3)

	>0 - <= 1.136 g	5	1023	0.8 (0.3,2.1)	0.8 (0.3,2.1)

	0 (Referent)	18	2922	1.0 (--------)	1.0 (--------)

					

Gastrointestinal	Any	11	1608	1.8 (0.8,4.2)	1.8 (0.7,4.4)

	>1.136 g (40 ug/L)	6	585	2.7 (1.0,7.4)	2.7 (0.9,7.6)

	>0 - <= 1.136 g	5	1023	1.3 (0.4,3.7)	1.3 (0.4,3.8)

	0 (Referent)	11	2915	1.0 (--------)	1.0 (--------)

					

Oral Clefts^&^	Any	5	1602	3.0 (0.7,13)	3.2 (0.7,15)

	>1.136 g (40 ug/L)	3	582	5.0 (1.0,25)	5.2 (0.9,30)

	>0 - <= 1.136 g	2	1020	1.9 (0.3,11)	1.8 (0.3,10)

	0 (Referent)	3	2907	1.0 (--------)	1.0 (--------)

					

Genitourinary	Any	11	1608	1.7 (0.7,3.8)	1.6 (0.6,3.8)

	>1.136 g (40 ug/L)	4	583	1.7 (0.5,5.2)	1.6 (0.5,5.0)

	>0 - <= 1.136 g	7	1025	1.7 (0.7,4.2)	1.6 (0.6,4.3)

	0 (Referent)	12	2916	1.0 (--------)	1.0 (--------)

					

Hypospadias^$^	Any Exposure	6	1603	1.8 (0.6,5.6)	1.4 (0.4,5.4)

	>1.136 g (40 ug/L)	2	581	1.7 (0.3,8.3)	1.5 (0.3,7.2)

	>0 - <= 1.136 g	4	1022	1.9 (0.5,6.8)	1.4 (0.3,6.5)

	0 (Referent)	6	2910	1.0 (--------)	1.0 (--------)

					

Musculoskeletal	Any	19	1616	0.9 (0.5,1.6)	0.9 (0.5,1.6)

	>1.136 g (40 ug/L)	10	589	1.4 (0.7,2.7)	1.5 (0.8,2.9)

	>0 - <= 1.136 g	9	1027	0.7 (0.3,1.4)	0.6 (0.3,1.3)

	0 (Referent)	37	2941	1.0 (--------)	1.0 (--------)

					

Respiratory	Any	0	1597	--------	--------

	>1.136 g (40 ug/L)	0	579	--------	--------

	>0 - <= 1.136 g	0	1018	--------	--------

	0 (Referent)	3	2905	--------	--------

					

Chromosomal	Any	3	1600	1.4 (0.3, 6.1)	1.4 (0.3,6.1)

	>1.136 g (40 ug/L)	0	579	--------	--------

	>0 - <= 1.136 g	3	1021	2.1 (0.5,9.6)	--------

	0 (Referent)	4	2908	1.0 (--------)	1.0 (--------)

					

Eye	Any	1	1598	--------	--------

	>1.136 g (40 ug/L)	1	580	--------	--------

	>0 - <= 1.136 g	0	1018	--------	--------

	0 (Referent)	7	2911	--------	--------

					

Ear	Any	0	1597	--------	--------

	>1.136 g (40 ug/L)	0	579	--------	--------

	>0 - <= 1.136 g	0	1018	--------	--------

	0 (Referent)	3	2907	--------	--------

					

Other	Any	2	1599	--------	--------

	>1.136 g (40 ug/L)	0	579	--------	--------

	>0 - <= 1.136 g	2	1020	--------	--------

	0 (Referent)	4	2908	--------	--------

*We attempted to calculate unadjusted GEE ORs if there were at least three exposed cases; in some instances the model did not converge and so the ORs are not available.

^+ ^Included among central nervous system defects

^&^Included among gastrointestinal defects

^$ ^Included among genitourinary defects

Among the nine children affected by neural tube defects, there were four exposed cases of anencephaly in three different families (crude prevalence 2.4/1,000) vs. no unexposed cases; one exposed case of spina bifida (crude prevalence 0.6/1,000) vs. three unexposed cases (crude prevalence 1/1,000); and one exposed case of Arnold-Chiari malformation (crude prevalence 0.6/1,000) vs. no unexposed cases.

Adjusted GEE odds ratios were fairly stable when we attempted to control for confounders one at a time among organ system subgroups with at least 20 cases. Adjusted odds ratios ranged from 0.8 to 1.0 for cardiac defects (crude GEE OR: 0.9), 1.6 to 2.0 for gastrointestinal defects (crude GEE OR: 1.8), 1.3 to 2.0 for genitourinary defects (crude GEE OR: 1.6), and 0.9 to 1.1 for musculoskeletal defects (crude GEE OR: 0.9). Confounders controlled in these analyses included those with apparent differences between the exposed and unexposed groups (Table 2), including calendar year of birth; maternal and paternal age; maternal cigarette smoking, alcoholic beverage consumption, and prior pregnancy losses; and child's gender.

The small number of affected children limited our ability to examine the organ system and diagnostic group associations for the presence of a dose-response relationship; however, we found that odds ratios for all gastrointestinal defects combined and oral clefts were further increased among children whose average monthly prenatal exposure was greater than 1.136 grams (Table 4). No dose-response relationship was observed for neural tube defects.

While we were able to validate only a small number of questionnaire reports against prenatal and obstetric records, we found excellent agreement between the information provided by the mothers and these records. For example, 92% of clinically recognized miscarriages, and 100% of live births noted in survey were reported in the medical record. There was also excellent agreement between the survey and medical record on gestational duration, birth weight, prenatal cigarette smoking, alcohol consumption, and multivitamin use. Furthermore, when we compared questionnaire and birth certificate data from all index children born in Massachusetts (n = 2,490), we found very good agreement on month and year of birth, mother's and father's age at the birth, birth weight, number of prior live births, and number of prior pregnancy terminations (including spontaneous and induced abortions).

In contrast, when we compared the mother's self-assessed exposure status to that derived from the EPANET assessment, we found that only 15% of mothers considered exposed by the EPANET assessment thought that their drinking water was contaminated, whereas 28% of these mothers thought that their water was not contaminated and 57% were unsure. Similarly, we found that 37% of mothers considered unexposed by the EPANET assessment thought that their drinking water was not contaminated while 9% thought that their drinking water was contaminated and 53% were unsure.

## Discussion

The results of this study suggest that prenatal exposure to PCE increases the risk of certain kinds of congenital anomalies. Prenatal exposure was associated with large increases in the risk of gastrointestinal defects (particularly oral clefts), neural tube defects (particularly anencephaly) and, modest increases in the risk of genitourinary defects (particularly hypospadias). No meaningful increases in risk were seen for cardiac, musculoskeletal and chromosomal anomalies, and there were too few exposed cases to estimate odds ratios for eye; ear, face, and neck; respiratory; and other anomalies. An exposure-response relationship was observed for oral clefts but not for neural tube or genitourinary defects.

Several limitations of this study influence the interpretation of these results. First, these findings results are likely affected by exposure misclassification. Because individual level exposure measurements were not available for the study period, we estimated historical PCE exposures using a leaching and transport model developed by Webler and Brown [15] that predicted the annual mass of PCE delivered to each residence. The model was applied to water distribution system conditions in 1980 and was assumed to be representative of the entire study period. Furthermore, information on water consumption and bathing habits could not be recalled by a sizable number of mothers and so we were unable to incorporate these behaviors into our exposure assessment and data analysis.

Thus, while results from two validation studies indicate good correlation between PCE concentrations in historical water samples and exposure estimates based on the original Webler-Brown algorithm (Spearman correlation coefficient = 0.48, p < 0.0001) [26], and exposure estimates based on the EPANET water distribution system modeling software (Spearman correlation coefficient = 0.65, P < 0.001) [27], non-differential misclassification of the exposure remains likely.

The study was also limited by low statistical power stemming from the small number of congenital anomalies reported by the mothers. While the small number also made it difficult to control for confounders, the irregular geographic pattern of PCE exposure made it unlikely that exposed and unexposed mothers differed on both known and unknown risk factors for congenital anomalies. In fact, comparison of available crude and adjusted analyses in the present analysis and prior analyses of the study population [11,12] provide evidence of limited or no confounding.

Still another limitation stems from the use of maternal reports as the source of information on the congenital anomalies. While comparison between the questionnaires and birth records suggest that study mothers were good reporters of pregnancy-related information, the births occurred many years before the study's data collection and the mothers had no training in teratology, and so it is likely that some anomalies, particularly minor ones, were not reported. Because pediatric records were unavailable, the presence of under-reporting could not be directly verified; however, its likelihood is supported by comparing the prevalence of anomalies in our study population with those observed in the 1960s Collaborative Perinatal Project (CPP), a prospective study of 50,000 women and their pregnancies. Using a comprehensive medical surveillance system, the CPP observed a prevalence of 45.3 malformations per 1,000 live- and still births [28] as compared to a prevalence of 33.5 per 1,000 in our study population. Surveillance data from the Metropolitan Atlanta Congenital Defects Program, and the CDC Birth Defects Monitoring Program during the 1970s and 1980s also support this notion [e.g., [29-32]].

While under-reporting likely affected the statistical stability of our findings, there is no evidence that it was more or less likely to occur among exposed mothers, because, as described in the results section, most mothers did not know their "true" exposure status. Thus, because the risk of congenital anomalies was small (<10%), the likely impact was a small bias towards the null [33].

Lastly, while non-participating mothers were younger and less educated than participating mothers, these differences were present for both exposed and unexposed non-participants, and so it is unlikely that selection bias influenced the current results. However, because birth certificates were used to identify study mothers, our results may not be generalizable to women who have never achieved a live birth.

Numerous animal experiments have observed teratogenic effects of prenatal exposure to PCE, TCE, and their metabolite trichloroacetic acid (TCA) that appear to be species- and dose-dependent. Increased rates of several types of malformations, including cardiac, ocular, and skeletal defects, have been seen in chick embryo cultures over a wide range of exposure levels, including doses as low as 1 umol [7,34,35]. Increased rates of malformations in the central nervous, musculoskeletal, and cardiovascular systems have also been observed among rats exposed to a wide range of TCE, PCE, and TCA exposures [8,36-39]. In fact, cardiac malformations were seen among offspring of pregnant rats who ingested drinking water with TCE levels as low as 250 ppb [39]. In contrast, no evidence of teratogenicity has been observed in experimental studies of exposed mice and rabbits [40,41].

Several epidemiological studies have also found associations between maternal occupational exposure to organic solvents and congenital anomalies [42]. A recent meta-analysis found a statistically significant association with major malformations (summary OR, 1.6; 95% CI, 1.2-2.3) [43]. However, these results are difficult to interpret regarding the risk associated with PCE exposure because a combination of many solvents were examined and because organ system-specific analyses were not conducted. Studies among dry cleaners exposed to PCE have found no increases in the risk of congenital anomalies, but small sample sizes limited their statistical power to examine rare outcomes like anomalies. The total number of cases in these studies ranged from three to thirty-eight [44-47].

In contrast, three drinking water studies have found positive associations for congenital anomalies. Goldberg et al. [9] found that the prevalence of cardiac anomalies in Tuscon, Arizona was three times higher among children of parents who had contact with TCE contaminated drinking water as compared to unexposed parents (p < 0.005). The contaminants present in the Tuscon Valley water supply included TCE (6 to 239 ug/dL), chromium (below the action level of 0.1 mg/L, and dichloroethylene (concentrations typically between 5-10% of trichloroethylene levels). Lagakos et al. [48] found that the offspring of women exposed to well water contaminated with TCE (267 ppb), PCE (21 ppb), trichlorotrifluoroethane (23 ppb) and dichloroethylene (28 ppb) in Woburn, Masssachusetts had an increased prevalence of eye and ear anomalies combined, and central nervous system, chromosomal, and oral cleft malformations combined. While many questions have been raised about this study, including the unusual malformation groupings [e.g., [49,50]], the results are consistent with the animal studies described above and an epidemiological study by Bove et al [10]. The latter New Jersey study found that PCE drinking water levels > 10 ppb were associated with a 3.5-fold increased risk of oral clefts (90% CI: 1.3 - 8.8), that TCE drinking water levels > 5 ppb were associated with a 2.2 fold increased risk of oral clefts (90% CI: 1.2 - 4.2), and that TCE levels > 10 ppb were associated with a 2.5-fold increased risk of neural tube defects (90% CI: 0.9 - 6.4). In contrast, a follow-up study in Woburn found no increases in the risk of cardiac, genital or musculoskeletal defects among children with prenatal exposure to contaminated well water [51]. While the authors concluded that the number of children with other defects was too small for meaningful analysis, they did observe a statistically unstable three to four-fold increased prevalence of eye anomalies based on four exposed cases. Reports of congenital anomalies were obtained from vital records and a more sensitive model of the water distribution system estimated prenatal exposures in the follow-up study.

Taken together, the results of the present and prior studies provide mounting evidence of an increased risk of oral clefts, and perhaps neural tube defects and other anomalies in relation to prenatal PCE exposure from drinking water. However, weaknesses in these studies, including the present one, make it important to confirm these findings in a follow-up investigation with a large number of affected children who are identified using medical or vital records. Because PCE remains a commonly used solvent and frequent contaminant of ground and drinking water supplies [6,52], it is important to understand its impact on the occurrence of congenital anomalies.

## Conclusion

Prior studies of prenatal exposure to solvents have found increases in the risk of congenital anomalies among exposed offspring, and so we undertook a retrospective cohort study to examine whether prenatal exposure to PCE-contaminated public drinking water influenced the occurrence of congenital anomalies in the Cape Cod region of Massachusetts. We found that children with prenatal exposure had increased risks of oral clefts and neural tube defects; however, an exposure-response relationship was observed for oral clefts but not for neural tube defects. These findings were limited by the small number of children with anomalies that were based on maternal reports. Therefore, we recommend that a follow-up investigation be conducted with a larger number of affected children who are identified by independent records.

## Abbreviations

AC: asbestos cement; DEP: Department of Environmental Protection; EPANET: publicly available software from the U.S. Environmental Protection Agency that models water flow through distribution systems; GIS: geographic information system; PCE: perchloroethylene, tetrachloroethylene; TCE: trichloroethylene; VL: vinyl lined.

## Competing interests

Dr. Ann Aschengrau currently serves as a plaintiff's consultant on a personal injury case involving exposure to tetrachloroethylene and trichloroethylene. Dr. David Ozonoff is editor-in-chief of Environmental Health but was not involved in the editorial handling of this manuscript. In addition, he, on occasion, testified in personal injury cases involving exposure to tetrachloroethylene and trichloroethylene. No such litigation is currently pending for Dr. Ozonoff. None of the other authors of this study have any past or present competing financial interests.

## Authors' contributions

AA designed the study, oversaw the data collection and analysis, and drafted the manuscript. JMW led the statistical analyses and collaborated on editorial issues. PAJ coded the malformation diagnoses and consulted on analytical and editorial issues. LGG conducted the exposure assessments and consulted on analytical and editorial issues. MRW conducted the epidemiological and statistical analyses and collaborated on editorial issues. TFW, VMV, and DMO collaborated on analytical and editorial decisions. All authors read and approved the final manuscript.
